# Ebselen reduces cigarette smoke‐induced endothelial dysfunction in mice

**DOI:** 10.1111/bph.15400

**Published:** 2021-02-27

**Authors:** Kurt Brassington, Stanley M. H. Chan, Huei Jiunn Seow, Aleksandar Dobric, Steven Bozinovski, Stavros Selemidis, Ross Vlahos

**Affiliations:** ^1^ School of Health & Biomedical Sciences RMIT University Bundoora Victoria Australia

**Keywords:** antioxidant, cardiovascular disease, chronic obstructive pulmonary disease, cigarette smoke, endothelium, lung inflammation, vascular dysfunction

## Abstract

**Background and Purpose:**

It is well established that both smokers and patients with COPD are at a significantly heightened risk of cardiovascular disease (CVD), although the mechanisms underpinning the onset and progression of co‐morbid CVD are largely unknown. Here, we explored whether cigarette smoke (CS) exposure impairs vascular function in mice and given the well‐known pathological role for oxidative stress in COPD, whether the antioxidant compound ebselen prevents CS‐induced vascular dysfunction in mice.

**Experimental Approach:**

Male BALB/c mice were exposed to either room air (sham) or CS generated from nine cigarettes per day, 5 days a week for 8 weeks. Mice were treated with ebselen (10 mg·kg^−1^, oral gavage once daily) or vehicle (5% w/v CM cellulose in water) 1 h prior to the first CS exposure of the day. Upon killing, bronchoalveolar lavage fluid (BALF) was collected to assess pulmonary inflammation, and the thoracic aorta was excised to investigate vascular endothelial and smooth muscle dilator responses ex vivo.

**Key Results:**

CS exposure caused a significant increase in lung inflammation which was reduced by ebselen. CS also caused significant endothelial dysfunction in the thoracic aorta which was attributed to a down‐regulation of eNOS expression and increased vascular oxidative stress. Ebselen abolished the aortic endothelial dysfunction seen in CS‐exposed mice by reducing the oxidative burden and preserving eNOS expression.

**Conclusion and Implications:**

Targeting CS‐induced oxidative stress with ebselen may provide a novel means for treating the life‐threatening pulmonary and cardiovascular manifestations associated with cigarette smoking and COPD.

**Abbreviations**
AECOPD: acute exacerbations of chronic obstructive pulmonary disease
BAL: bronchoalveolar lavage
BALF: bronchoalveolar lavage fluid
COPD: chronic obstructive pulmonary disease
CS: cigarette smoke
CVD: cardiovascular disease
eNOS/NOS3: endothelial NOS
Gpx‐1: GSH peroxidase‐1
MS: mechanosensitive
NOX: nicotinamide adenine dinucleotide phosphate oxidase
RNS: reactive nitrogen species
VECs: vascular endothelial cells

**What is already known**

- Co‐morbid cardiovascular disease is the major cause of death in COPD patients.
- Oxidative stress drives COPD but its contribution to cardiovascular outcomes in COPD remains unknown.

**What does this study add**

- Cigarette smoke‐induced inflammation and oxidative stress cause vascular endothelial dysfunction.

**What is the clinical significance**

- Targeting oxidative stress can treat both the pulmonary and cardiovascular manifestations associated with COPD.

## 1 INTRODUCTION

Chronic obstructive pulmonary disease (COPD) is a major incurable global health burden and is currently the fourth largest cause of death in the world (The World Health Organisation, 2018). Approximately 50% of COPD patients will die from a cardiovascular event (Sin & Man, 2005), and consequently, the pathobiological mechanisms linking COPD to cardiovascular disease are now an area of intensive research. Each puff of cigarette smoke contains >10^16^ free radicals per puff, driving oxidative stress and tissue damage (Bartalis et al., 2007). Such oxidative stress and inflammation alter pulmonary blood vessel structure, through driving vascular remodelling, and promoting arterial stiffness and atherosclerosis (Sin et al., 2006). Vascular tone is controlled by vasoactive substances such as NO and PGs, and their secretion can be maintained by circulating oxygen levels (Chan & Vanhoutte, 2013). Under hypoxic conditions, as those seen in COPD, dysregulation of this vascular homeostatic balance occurs, due to oxidative damage to the vascular endothelial cells (VECs) leading to impaired NO production, thereby promoting endothelial dysfunction (Chan & Vanhoutte, 2013). NO is a key vasodilator produced by endothelial NOS (eNOS). Under normal physiological conditions, increased sheer stress on the vascular endothelium stimulates mechanosensitive ion channels, triggering a rapid influx of Ca^2+^ into the cytoplasm of the VECs. This increases eNOS activity via myoendothelial gap junctions, that transmit vasodilatory NO signals to the underlying smooth muscle cells. VECs appear to be sensitive to oxidative damage, which may be the result of the conversion of NO to peroxynitrite (ONOO^−^) in the presence of the harmful ROS; superoxide anion (O_2_^−^) that ultimately reduces vascular NO bioavailability (Endemann & Schiffrin, 2004; Kolluru et al., 2012; Tabit et al., 2010). An altered oxidative balance in VECs has been demonstrated to favour cardiovascular events such as atherosclerosis, myocardial infarction (MI) and stroke (Brassington et al., 2019; Endemann & Schiffrin, 2004; Kolluru et al., 2012; Tabit et al., 2010).

Although cardiovascular co‐morbidities are the largest cause of mortality in COPD, the detrimental effects of CS and its associated oxidative stress on the systemic vasculature remain largely unknown. Given the deleterious role of oxidative stress in COPD, antioxidant treatment may be a viable therapeutic approach to treat the cardiovascular manifestations associated with this disease. We have previously shown that the antioxidant ebselen (2‐phenyl‐1,2‐benzisoselenazol‐3(2H)one), an organoselenium GSH peroxidase (Gpx) mimetic, inhibits CS‐induced lung inflammation in mice (Duong et al., 2010). Ebselen treatment may also be an effective therapeutic in chronic diseases such as atherosclerosis, thrombosis, and stroke where oxidative stress and inflammation play a crucial role (Azad & Tomar, 2014; Sarker et al., 2003; Sarma & Mugesh, 2008; Takasago et al., 1997). Moreover, studies have shown that Gpx‐1 deficient mice have enhanced pulmonary inflammation (Duong et al., 2010), as well as worsened cardiovascular outcomes including a larger infarct volume following ischaemic stroke (Crack et al., 2001; Duong et al., 2010), suggesting that Gpx‐1 (or compounds which mimic its actions, such as ebselen) may exhibit protective effects.

Of interest, Gpx‐1 activity is elevated in smokers, as a potential mechanism to counteract the harmful oxidative stress. However, Gpx‐1 is severely depleted in the lungs of COPD patients, resulting in an overstated inflammatory response and oxidative burden (Kluchova et al., 2007; Santos et al., 2004; Tkacova et al., 2007; Vlahos et al., 2010). A study by Chew et al. has shown therapeutic effects of ebselen in Gpx‐1 knockout mice, with the study finding that synthetic repletion of Gpx activity in these diabetic mice produced athero‐protective effects in vivo (Chew et al., 2010). Exogenous repletion of Gpx‐1 with compounds like ebselen may have therapeutic potential in not only treating the pulmonary manifestations of COPD but perhaps its cardiovascular co‐morbidities.

In the present study, we investigated whether chronic CS exposure in a preclinical mouse model of COPD impaired vascular function and whether ebselen could prevent CS‐induced vascular dysfunction in mice.

## 2 METHODS

### 2.1 Animals

All animal care and experimental procedures were conducted in accordance with the Australian Code of Practice for the Care of Experimental Animals and with the approval of the RMIT University Animal Ethics Committee (Animal Ethics Application Number 1521). Animal studies are reported in compliance with the ARRIVE guidelines (Percie du Sert et al., 2020) and with the recommendations made by the British Journal of Pharmacology (Lilley et al., 2020). Seven‐week‐old male Balb/c mice (~20 g body weight) were obtained from the Animal Resource Centre Pty. Ltd (Perth, Australia). Mice were housed in micro‐isolator cages (Able Scientific, Australia) at 21°C with ad libitum access to water and standard mouse chow (Glen Forest Speciality Foods, Australia).

### 2.2 Cigarette smoke exposure and ebselen treatment

Mice were placed into an 18‐L Perspex chamber (The Plastic Man, Huntingdale, Victoria, Australia) in a standard fume cabinet (Aircare Extraction Systems LTD, Clayton, Victoria, Australia) and exposed to cigarette smoke generated from 9 Winfield Red Cigarettes (total particulate matter of 419 mg·m^−3^, 16 mg or less of tar, 1.2 mg or less of nicotine and 15 mg or less of CO, Philip Morris, Moorabbin, Australia) for 5 days a week for 8 weeks. Mice were exposed to CS (*n* = 10 per treatment group) delivered three times per day with three cigarettes delivered at 9 a.m., 12 noon, and 3 p.m., over a 1 h time‐period. CS was generated in 60 ml tidal volumes over 10 s, via a timed draw‐back to mimic normal smoking inhalation and burn rates. We have previously shown that this CS exposure protocol in Balb/C mice replicates key clinical traits of early stage COPD in humans, including lung inflammation and pathology (mucus hypersecretion and impaired lung function), increased lung and systemic oxidative stress and co‐morbidities including skeletal muscle wasting (Austin et al., 2016; Chan et al., 2019; Vlahos & Bozinovski, 2014). Thus, this model provides a robust and clinically relevant platform to test therapies for COPD and its co‐morbidities. Sham‐exposed mice are placed into an identical 18‐L Perspex chamber but do not receive cigarette smoke. Mice were weighed every second day (prior to initial CS exposure) up to and including the end of the experiment. For the ebselen treatment studies, mice were administered 10 mg·kg^−1^ of ebselen (Sapphire Bioscience, Australia) prepared in 5% w/v CM‐cellulose in water (Sigma‐Aldrich, USA) or vehicle treated with 5% CM‐cellulose in water alone. Treatments were administered via oral gavage once daily, 1 h prior to the initial CS exposure.

### 2.3 Bronchoalveolar lavage and lung collection

Animals were killed at the end of the experimental protocol via intraperitoneal injection of sodium pentobarbitone (240 mg·kg^−1^; Virbac, NSW, Australia). Lungs were then lavaged in situ via a surgical tracheotomy with 0.4 ml of chilled PBS initially followed by 0.3 ml PBS three times, with ~1 ml of BAL fluid (BALF) retrieved per mouse as previously described (Vlahos et al., 2006). Total viable cell numbers in the BALF were determined using 50 μl of BALF diluted with 50 μl of acridine orange/ethidium bromide (AO/EB) (Invitrogen, USA). Cell counting was carried out using a standard Neubauer haemocytometer, under fluorescent light on an Olympus BX53 microscope (Olympus, Japan). Right ventricular perfusion with 6–7 ml of PBS was then performed to clear whole lungs from blood, and the lungs then excised, rinsed in PBS, snap‐frozen in liquid nitrogen and stored at −80°C until required.

### 2.4 Differential cell counting

To differentiate the various cell populations in the BALF, cytocentrifuged cell preparations (Shandon Cytospin 3, 18.06 g, 10 min) were used, with ~5 × 10^4^ cells per slide. Dried cytospots were subjected to a Shandon Kwik‐Diff® Fixative (Thermo Fischer Scientific, USA), followed by Hemacolour® eosin and thiazine differential stains (Merck, USA) as outlined in the manufacturer's instructions. Cell types (i.e. macrophages, neutrophils, and lymphocytes) were identified according to standard morphological criteria, using the above‐mentioned microscope, with at least 500 cells counted per slide.

### 2.5 Quantitative real‐time PCR (RT‐qPCR)

Total RNA was extracted from approximately 10 mg of whole lung tissue using a RNeasy® Mini Kit (Qiagen, Germany). Isolated mRNA was then reverse transcribed with a High Capacity RNA‐to‐cDNA kit (Thermo Fisher Scientific, USA). Real‐time PCR reactions were performed using Thermo Fisher Scientific pre‐developed Taqman assay reagents in triplicate using GAPDH as the internal housekeeping control. Through utilisation of the threshold cycle (C_t_) value which is the PCR cycle number out of 40 at which the fluorescence signal measured exceeds the calculated background threshold, indicative of amplification of the target sequence value, which is proportional to the number of target copies within the sample and converting this result to the threshold cycle time (∆∆CT). mRNA expression levels can then be quantified and referenced against GAPDH, allowing comparisons between treatment groups to be made.

### 2.6 Vascular reactivity

To assess the effect of both CS‐exposure and ebselen treatment on vessel function, the thoracic aorta was excised from each mouse and all perivascular fat removed. Vessels were placed into carbogen‐bubbled (95% O_2_, 5% CO_2_) cold Krebs buffer (composition in mmol·L^−1^: NaCl 119, KCl 4.7, MgSO_4_ 1.17, NaHCO_3_ 25, KH_2_PO_4_ 1.18, CaCl_2_ 2.5, glucose 5.5). The aortae were cut into four 2 mm rings and mounted onto the pins of the myograph system (Danish Myo Technology A/S, Model 610 M) with the resting tension increased to 5 mN which was determined to give an effective arterial wall pressure of ~100 mmHg (13.3 kPa) thus mimicking in vivo conditions. Following a 30‐min equilibration, aortic rings were exposed to 0.5 × 10^−3^ M of the TxA_2_ agonist U46619 (Cayman Chemical, USA) to induce the maximal vascular contraction, defined as 100%. In each ring, both endothelial integrity and smooth muscle function were assessed using cumulative doses of ACh (1 × 10^−8^M to 1 × 10^−5^M) (Thermo Fisher Scientific, USA) and sodium nitroprusside (SNP, 1 × 10^−8^M to 1 × 10^−5^M) (Thermo Fisher Scientific, USA) respectively in sub‐maximally contracted aorta (50–60% of maximal U46619 contraction) with all experiments run in duplicate and compared to sham or sham + vehicle treated mice.

### 2.7 Immunohistochemical staining for endothelial NOS and oxidative stress

Vascular expression of endothelial NOS was quantified using eNOS/NOS3 antibody (1:100 dilution, Thermo Fisher Scientific, USA), which is the key NO generating enzyme within the vascular endothelium. Vascular oxidative stress was measured using 3‐nitrotyrosine (3‐NT) (1:100 dilution, Thermo Fisher Scientific, USA) as this is a specific marker for peroxynitrite production, the direct product of the reaction between superoxide anion and NO. The excised thoracic aorta was fixed in 4% paraformaldehyde prior to sucrose saturation. The aortae were then paraffin embedded and 4 μM sections were cut. Aortic sections were subject to standard deparaffinisation and rehydration, followed by antigen retrieval (in 10 mM citric acid, 0.05% Tween 20, pH 6.0) and blocking (blocking buffer; 10% horse serum, 10% FBS, 2% Triton‐X, 1× PBS to 50 ml for 1 h). Sections were then incubated overnight with either eNOS of 3‐NT primary antibodies at 4°C. Excess primary antibodies were removed by washing, the sections were then incubated at room temperature with the fluorescently labelled secondary antibody, Alexa 488 (1:200 dilution, Thermo Fisher Scientific, USA), then cover slipped using Fluoromount‐G™, with DAPI (Thermo Fisher Scientific, USA) prior to imaging on an Olympus slide scanner VS120‐SS (Olympus, Japan), using Olympus VS‐ASW 2.9 imaging software (Olympus, Japan). The expression of eNOS and 3‐NT was quantified in the endothelial layer of the aortae using Olympus cellSens Dimension™ desktop software, calculating Object Area Fraction ROI (%) (version 1:18, Olympus Corporation). The immuno‐related procedures used comply with the recommendations made by the *British Journal of Pharmacology*.

### 2.8 Data and statistical analysis

The data and statistical analysis comply with the recommendations of the *British Journal of Pharmacology* on experimental design and analysis in pharmacology. All data are presented as mean ± SEM unless otherwise stated. Statistical differences between treatments were determined by ANOVA followed by Tukey's multiple comparison post hoc tests where appropriate. One‐way ANOVA were used for three of more unmatched groups. Two‐way ANOVA were used to analyse data when response was influenced by two categorized factors of interest. For comparisons between two groups of results, regular unpaired *t*‐tests were used to generate the *P* value. All statistical analyses were performed using GraphPad Prism™ for Microsoft Windows® (Versions 8, Graphpad software®, USA) where *P* < .05 was accepted as significant for all cases. This study was designed to generate groups of equal size, using randomisation and blinded analysis, with statistical analysis undertaken only for studies where each group size was at least *n* = 5, with group size being the number of independent values not technical replicates.

### 2.9 Materials

The suppliers of the following compounds are as follows: Winfield red cigarettes (Phillip Morris, Australia); ebselen (Sapphire Bioscience, Australia); CM‐cellulose (Sigma‐Aldrich, USA); sodium pentabarbitone (Virbac, Australia); acridine orange/ethidium bromide (Invitrogen, USA); Kwik‐Diff® reagent 1, fixative (Thermo Fisher Scientific, USA); Hemacolour® rapid staining of blood smear (eosin solution) (Merck, USA); Hemacolour® rapid staining of blood smear (thiazine solution) (Merck, USA); RNeasy Mini Kit (Qiagen, Germany); High Capacity RNA‐to‐cDNA kit (Thermo Fisher Scientific, USA); pre‐developed TaqMan primers to: TNF‐α (ID: Mm00443258_m), IL‐6 (ID: Mm00446190_m1), NOX‐2/CYBB (ID: Mm01287743_m1), GPx‐1 (ID: Mm00656767_g1) (Thermo Fisher Scientific, USA); U46619 (Cayman Chemical, USA); ACh (Thermo Fisher, USA); sodium nitroprusside (Thermo Fisher, USA); eNOS/NOS3 RRID: AB_2533121 (Thermo Fisher Scientific, USA), 3‐NT RRID: AB110282 (Abcam, USA); Goat anti‐mouse IgG (H + L) secondary antibody, Alexa Flour Plus 488 RRID: AB_2633275 (Thermo Fisher Scientific, USA); Fluoromount‐G™, with DAPI (Thermo Fisher Scientific, USA).

### 2.10 Nomenclature of targets and ligands

Key protein targets and ligands in this article are hyperlinked to corresponding entries in http://www.guidetopharmacology.org, and are permanently archived in the Concise Guide to PHARMACOLOGY 2019/20 (Alexander et al., 2019).

## 3 RESULTS

### 3.1 Chronic CS exposure causes endothelial dysfunction in mouse thoracic aorta

**FIGURE 1:**
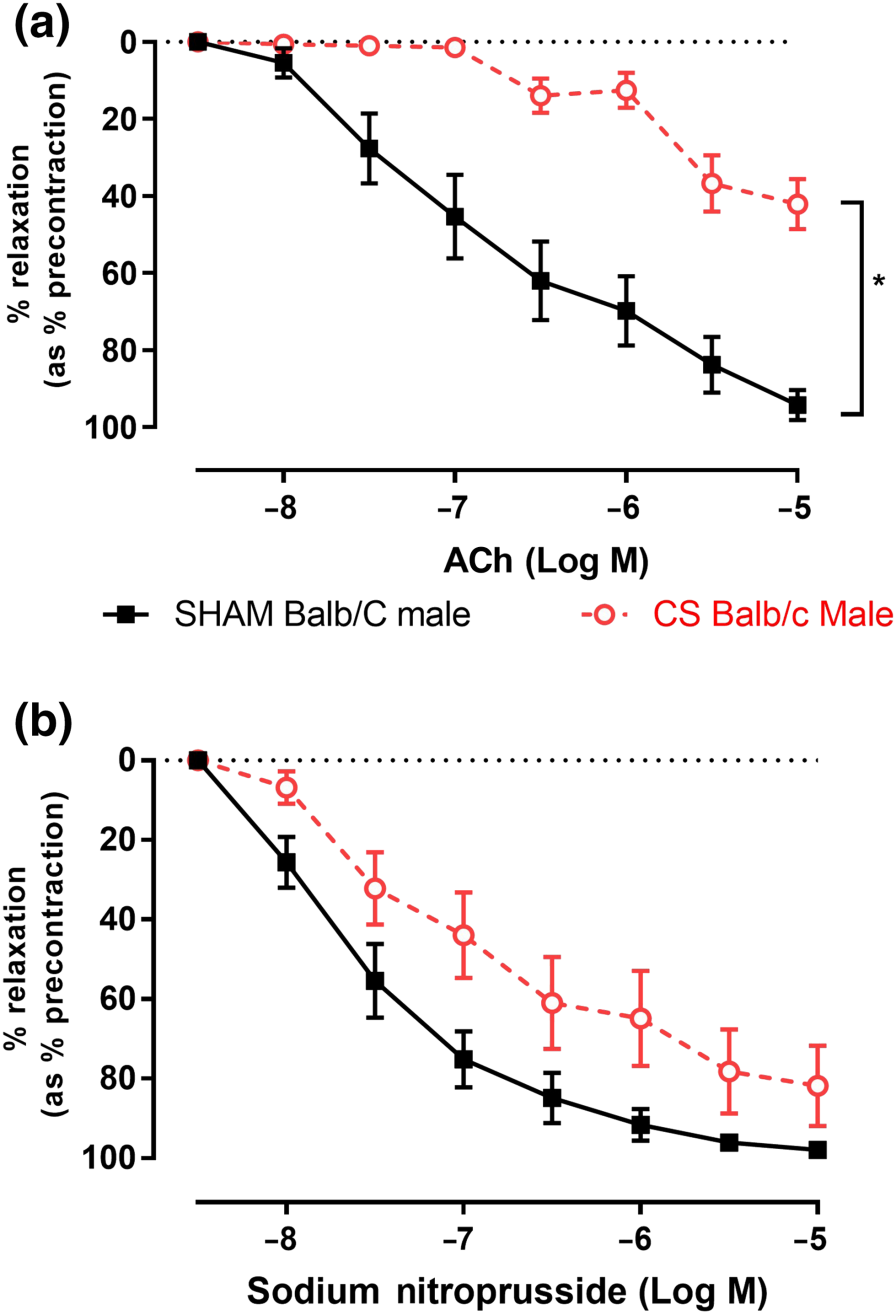
CS exposure causes endothelial dysfunction in the thoracic aorta. Cumulative concentration response curves to (a) ACh and (b) sodium nitroprusside (1 × 10^−8^M to 1 × 10^−5^M) to assess both endothelial‐dependent and smooth muscle‐dependent vasodilatory responses in mouse thoracic aorta (*n* = 8 per group) following either chronic CS or sham exposure, respectively. Results are expressed as mean percentage relaxation relative to precontraction ± SEM. *P < .05, significantly different as indicated; two‐way ANOVA with Tukey's multiple comparisons

The effect of chronic CS exposure on vascular function was examined to establish if the enhanced oxidative stress and inflammation arising from smoking has detrimental effects on blood vessel function in vivo. ACh (10^−8^ to 10^−5^ M) caused an ~90% maximal relaxation (R_max_) of U46619‐precontracted thoracic aorta obtained from sham‐exposed mice (Figure 1a). However, in thoracic aorta taken from CS‐exposed mice, endothelial‐dependent vasodilatory responses were significantly impaired (~40% R_max_), demonstrating that chronic CS exposure causes endothelial dysfunction. Sodium nitroprusside (10^−8^ to 10^−5^ M) caused an ~90–95% maximal relaxation of aortae taken from both sham and CS‐exposed mice with no significant difference observed between groups (Figure 1b), suggesting CS exposure has no effect on vascular smooth muscle function.

### 3.2 CS exposure drives pulmonary immune cell infiltration, pro‐inflammatory and oxidative stress gene expression

**FIGURE 2:**
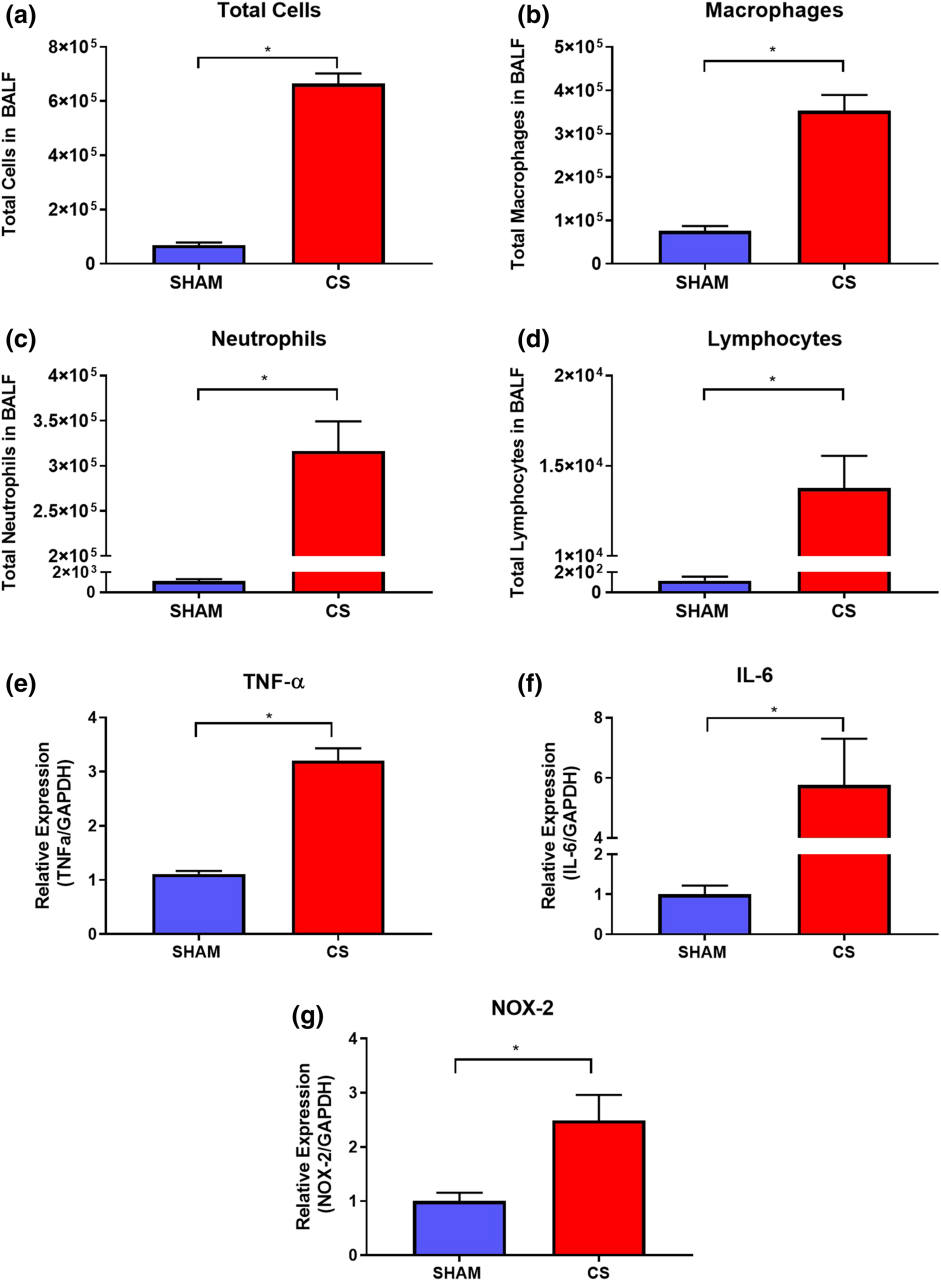
Chronic CS exposure increases BAL fluid cellularity and enhances both lung pro‐inflammatory and oxidative stress mediator gene expression. The lungs of mice exposed to CS were lavaged for the assessment of total cells (a), macrophages (b), neutrophils (c), and lymphocytes (d) (*n* = 10). Whole lungs excised from mice were then used to measure mRNA expression by RT‐qPCR of TNFα (e) (*n* = 10), IL‐6 (f) (*n* = 10), and NOX‐2 (g) (*n* = 9). Gene expression data are expressed as fold change relative to the sham group. All data are expressed as mean + SEM. *P < .05, significantly different as indicated; Student's unpaired *t*‐test

CS exposure caused a significant increase in the total number of immune cells infiltrating the lungs when compared to sham‐exposed control mice (Figure 2a). This increase in total cell number was attributed to a significant increase in macrophages, neutrophils, and lymphocytes (Figure 2b–d). To better understand the mechanism(s) underlying the increased BALF inflammation, the pulmonary expression of both pro‐inflammatory and oxidative stress genes was examined. CS exposure caused a significant increase in mRNA expression of the pro‐inflammatory cytokines TNF‐α (2.2‐fold) and IL‐6 (4.7‐fold), as well as the key oxidative stress enzyme NOX‐2 (1.5‐fold; Figure 2f,g).

### 3.3 Endothelial NOS expression is down‐regulated due to increased vascular ROS in COPD

**FIGURE 3:**
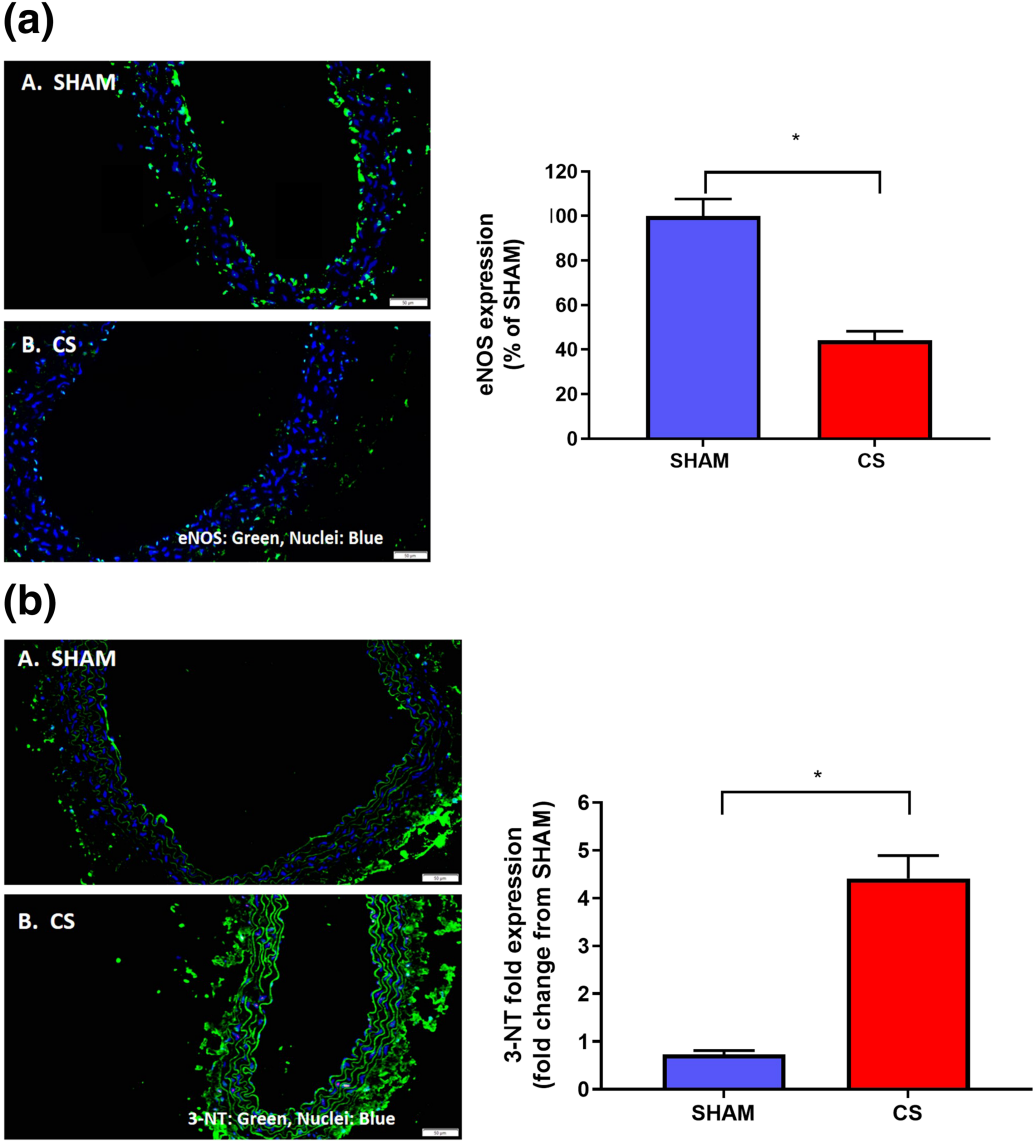
Immunofluorescent staining of endothelial NOS and 3‐nitrotyrosine in the thoracic aorta of mice exposed to 8 weeks of CS or room air. Immunofluorescent quantification of eNOS and 3‐NT expression in either sham or 8‐week CS‐exposed mice. Green staining indicates the presence of eNOS (a) or 3‐NT (b) and blue staining denotes the nuclear counterstain, DAPI. Representative photographs of immunofluorescent staining in (a) sham‐exposed or (b) CS‐exposed mice. After normalisation to the relevant negative control, the expression of eNOS (*n* = 5–6 mice per group) and 3‐NT (*n* = 5 mice per group) are presented as either percentage (%) change of or fold change from the sham‐treated group. Scale bar represents 50 μM. Data are expressed as mean + SEM. *P < .05, significantly different as indicated; Student's unpaired *t*‐test

Given that chronic CS exposure causes endothelium‐dependent vascular dysfunction, we then went on to investigate the potential underlying mechanism driving this impaired vascular function. Endothelial expression of the key vascular tone regulator eNOS and a marker of oxidative stress, 3‐nitrotyrosine (3‐NT), were quantified. CS exposure significantly reduced expression of eNOS by ~70% (Figure 3a). In addition, the level of 3‐NT expression was significantly up‐regulated by ~3.2‐fold following CS exposure (Figure 3b), indicative of enhanced vascular oxidative stress which may be responsible for the reduced expression of eNOS.

### 3.4 Ebselen prevents CS‐induced endothelial dysfunction

**FIGURE 4:**
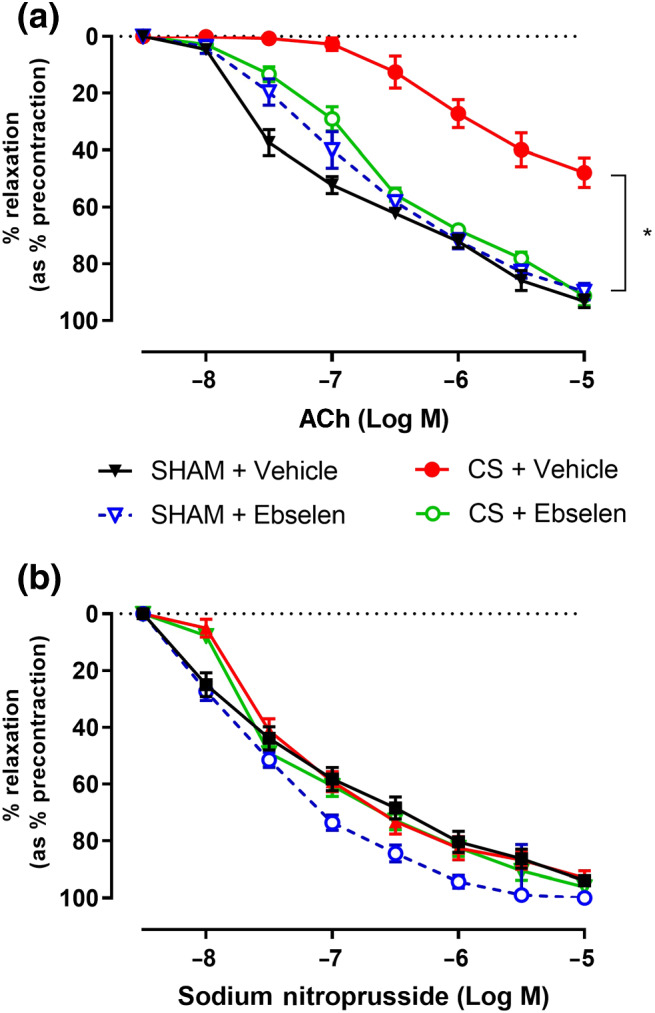
Ebselen prevents CS‐induced endothelial dysfunction. Cumulative concentration response curves to ACh (a) and sodium nitroprusside (b) (1 × 10^−8^M to 1 × 10^−5^M) to assess endothelial and smooth muscle‐dependent vasodilation in mouse thoracic aorta (*n* = 6) following either chronic CS or sham exposure in either ebselen or vehicle‐treated mice, respectively. Results are expressed as mean percentage relaxation relative to pre‐constriction ± SEM. *P < .05, significantly different as indicated; two‐way ANOVA with Tukey's multiple comparisons

As chronic CS exposure caused significant lung inflammation as well as heightened lung and vascular oxidative stress, we sought to investigate the effect of antioxidant treatment on vascular endothelial function. Sham + Veh‐treated mice showed an ~90% R_max_, while CS + Veh‐treated mice showed an ~40% R_max_ to ACh (Figure 4a), confirming CS‐induced endothelial dysfunction as also demonstrated in Figure 1. CS‐exposed mice treated with ebselen were completely protected from the CS‐induced endothelial dysfunction, as shown by the maximal relaxation of ~90% to ACh. In addition, ebselen did not affect vascular endothelial function in sham‐exposed mice (Figure 4a), suggesting that its protective effects are specific to CS exposure. As shown in Figure 1, smooth muscle relaxant responses to SNP were unaltered, regardless of CS exposure or ebselen treatment (Figure 4b).

### 3.5 Ebselen reduces pulmonary immune cell infiltration but has no effect on pro‐inflammatory and oxidative stress gene expression

**FIGURE 5:**
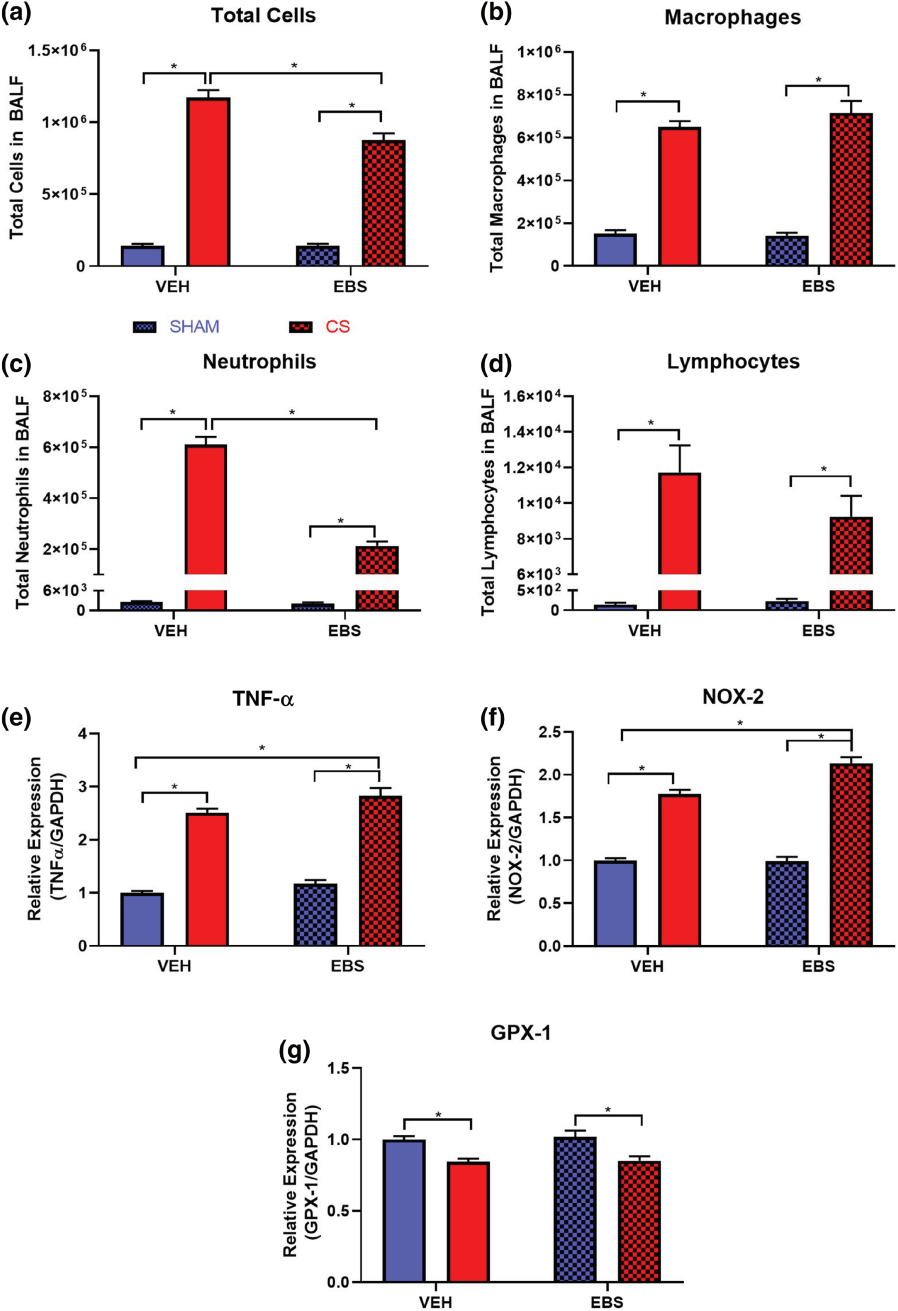
Effect of chronic CS and ebselen treatment on BAL fluid cellularity and pulmonary pro‐inflammatory and oxidative stress gene expression. The lungs of mice exposed to CS were lavaged for the assessment of total cells (a), macrophages (b), neutrophils (c), and lymphocytes (d) (*n* = 10). Whole lungs excised from mice were then used to measure mRNA expression by RT‐qPCR of TNF‐α (e), NOX‐2 (f), and GPX‐1 (g) (*n* = 10). Responses are expressed as fold change relative to the sham + vehicle‐treated group, post normalisation to GAPDH (housekeeping gene). All data are expressed as mean + SEM. *P < .05, significantly different as indicated; two‐way ANOVA and Tukey's post hoc analysis

Given that ebselen was able to protect against CS‐induced vascular dysfunction, we next sought to determine the effect of ebselen on CS‐induced lung inflammation. Consistent with Figure 2, CS caused a significant increase in BALF total cells, macrophages, neutrophils, and lymphocytes (Figure 5a–d). Interestingly, ebselen significantly reduced CS‐induced increases in BALF total cell and neutrophil counts but not macrophage and lymphocyte numbers (Figure 5a–d). Moreover, while CS exposure increased TNF‐α and NOX‐2 mRNA expression in the lungs, no detectable attenuations were found after ebselen administration (Figure 5e,f). Ebselen administration also appears to be ineffective in preserving lung Gpx‐1 mRNA expression against CS exposure (Figure 5g) but rather mimics its antioxidant activity.

### 3.6 Ebselen diminishes CS‐induced vascular oxidative stress and down‐regulation of eNOS

**FIGURE 6:**
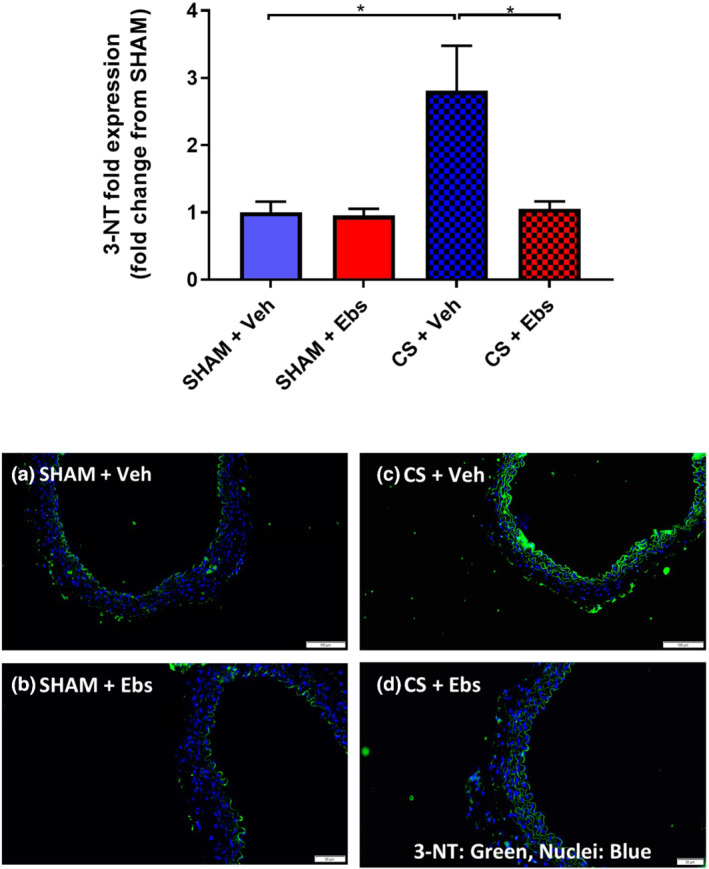
Immunofluorescent staining of 3‐nitrotyrosine in the thoracic aorta of mice exposed to chronic CS and treated with ebselen. Immunofluorescent quantification of 3‐NT expression in either sham or 8‐week CS‐exposed mice that were treated with either vehicle or ebselen. Green staining indicates the presence of 3‐NT specific and blue staining denotes the nuclear counterstain, DAPI. Representative photographs of immunofluorescent staining in (a) sham‐exposed vehicle treated (*n* = 5), (b) sham‐exposed ebselen treated (*n* = 5), (c) CS‐exposed vehicle treated (*n* = 5), and (d) CS‐exposed ebselen treated mice (*n* = 6). After normalisation to the relevant negative control, the expression of 3‐NT is presented as fold change from the sham vehicle‐treated group. Scale bar represents 50 μM. Data are expressed as mean + SEM. *P < .05, significantly different as indicated; two‐way ANOVA with Tukey's multiple comparisons

**FIGURE 7:**
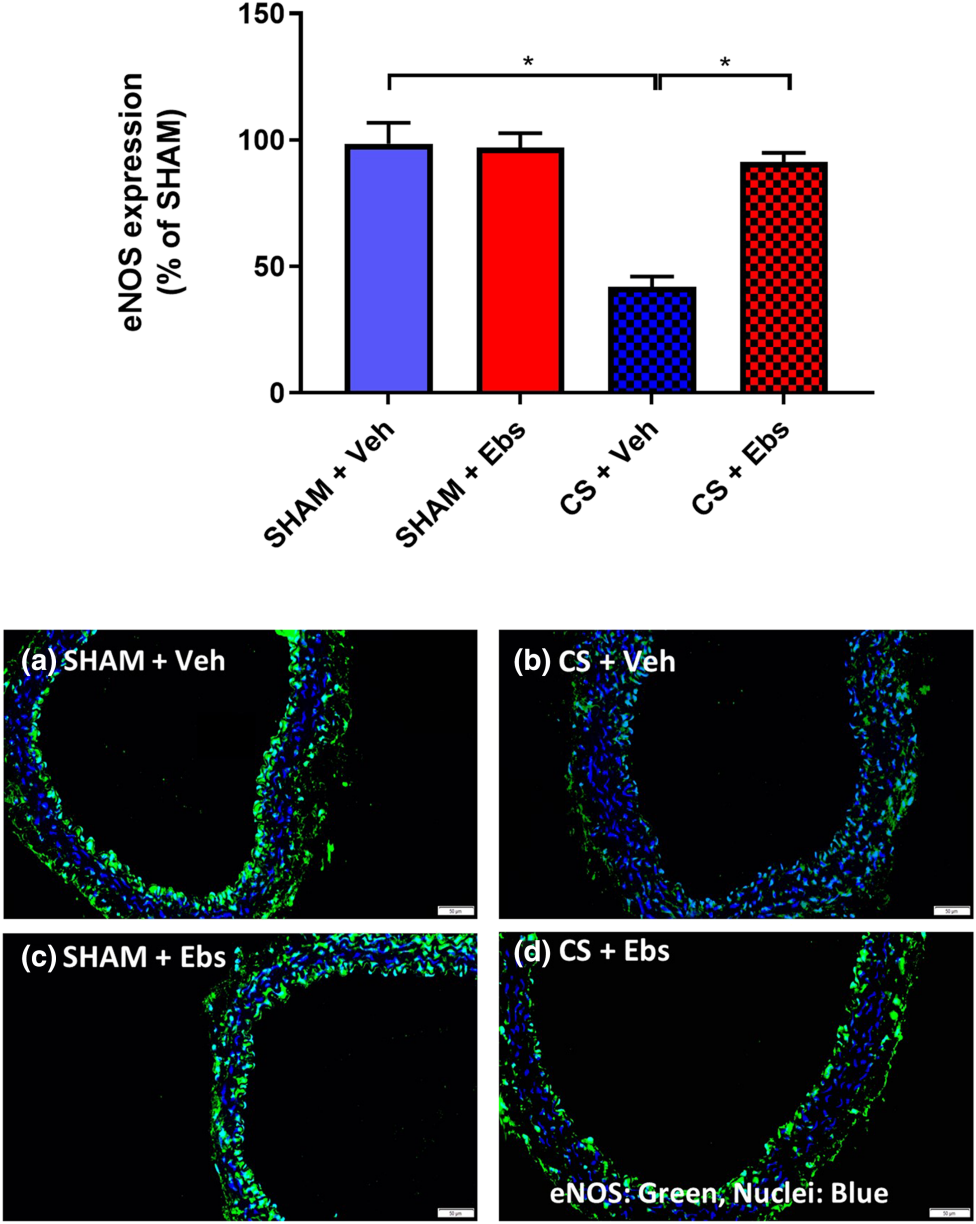
Immunofluorescent staining of endothelial NOS in the thoracic aorta of mice exposed chronically to CS and treated with ebselen Immunofluorescent staining of eNOS in either sham or 8‐week CS‐exposed mice with or without ebselen administration. Green staining detects the presence of eNOS and blue staining denotes the nuclear counterstain, DAPI. Representative photographs of immunofluorescent staining in (a) sham‐exposed vehicle treated (*n* = 5), (b) sham‐exposed ebselen treated (*n* = 5), (c) CS‐exposed vehicle treated (*n* = 7), and (d) CS‐exposed ebselen treated mice (*n* = 7). After normalisation to the relevant negative control, the expression of eNOS is presented as percentage (%) change of the sham vehicle‐treated group. Scale bar represents 50 μM. Data are expressed as mean ± SEM. *P < .05, significantly different as indicated; two‐way ANOVA with Tukey's multiple comparisons

We next sought to define the underlying mechanism by which ebselen prevented CS‐induced endothelial dysfunction. Mice chronically exposed to CS had increased vascular endothelial oxidative stress as measured by 3‐NT expression (Figure 6). However, ebselen administration significantly reduced CS‐induced increases in 3‐NT expression to baseline sham levels (Figure 6). Moreover, ebselen treatment completely prevented the down‐regulation of eNOS as a result of CS‐exposure (Figure 7). Collectively, these findings suggest that ebselen reduced CS‐induced vascular oxidative stress and subsequently prevented the down‐regulation of eNOS within the thoracic aorta.

## 4 DISCUSSION

In the present study, the mechanisms underlying systemic endothelial dysfunction, the key driver of co‐morbid CVD‐associated mortality are revealed and the effects of CS exposure on blood vessel function in our preclinical murine model of COPD were assessed. We found that 8 weeks of CS exposure caused significant pulmonary inflammation and oxidative stress in mice. Pulmonary immune cell infiltration is believed to be the underlying factor driving increased NOX‐2 expression and ROS formation. Studies from our group have shown that mice treated with influenza A virus had increased levels of ROS production in the lungs as a consequence of increased pulmonary inflammation (macrophages and neutrophils) and NOX‐2 expression (To et al., 2017; Vlahos et al., 2011; Vlahos & Selemidis, 2014). In this study, we also showed increased NOX‐2 expression in the lung which is presumably a result of increased pulmonary macrophage and neutrophil numbers in response to CS exposure. We also found a significant increase in the expression of the pro‐inflammatory mediators TNF‐α and IL‐6 in response to CS, which is consistent with our previously published work (Hansen et al., 2013; Vlahos et al., 2006). TNF‐α is largely secreted by stimulated macrophages (e.g. in response to CS), driving the inflammatory response and intracellular ROS production, while down‐regulating antioxidant activity (Mukhopadhyay et al., 2006). IL‐6 has also been implicated in the pathophysiology of pulmonary diseases. Thus, these pro‐inflammatory mediators may contribute to pulmonary inflammation and reduced lung function observed in COPD patients (Rincon & Irvin, 2012).

It was clear from the present study that CS exposure significantly impaired vasodilation of mouse thoracic aorta to ACh and that this was specifically attributed to endothelial dysfunction without affecting smooth muscle function. This CS‐induced endothelial dysfunction may be a critical link between the heightened risk of CVD and related mortality that claims the lives of ~50% of COPD patients, as well as current and ex‐smokers. It has been well characterized in models of diabetes mellitus (DM) that increased vascular oxidative stress is the key driver of endothelial dysfunction seen in diabetic complications, as a result of enhanced ROS production and reduced NO bioavailability in the vascular wall (de Haan & Cooper, 2011; Kolluru et al., 2012; Rask‐Madsen & King, 2007; Shenouda et al., 2011; Tabit et al., 2010). Increased oxidative stress in patients with COPD may lead to post‐translational modification of eNOS and increase vascular oxidative stress, thereby resulting in endothelial dysfunction, similar to that observed in DM (de Haan & Cooper, 2011).

Having shown that CS causes endothelium‐dependent vascular dysfunction, we next investigated whether this was attributed to changes in eNOS expression. We found that CS caused an ~60% reduction in the expression of eNOS, which would likely lead to a drastic reduction in the production of the key vasodilator NO and reduced NO bioavailability, a typical feature of CVD. With studies like that of de Hann et al. showing that eNOS can undergo oxidative modification under highly oxidative environments (de Haan & Cooper, 2011), vascular expression levels of peroxynitrite were therefore analysed using 3‐NT, which specifically detects the reaction of superoxide anions and NO to yield ONOO^−^. In agreement with this, the present study also found enhanced vascular oxidative burden following exposure to CS, which in turn may promote endothelial dysfunction via post‐translational modifications of eNOS and decreasing the bioavailability of NO.

Given the significant role of oxidative stress in COPD and this study, we tested if the administration of ebselen, an antioxidant drug which has shown promising results in the context of DM‐induced vascular complications through its free radical scavenging activity, could prevent CS‐induced vascular dysfunction. Moreover, de Haan and Cooper (2011) have proposed that deficiencies in the antioxidant enzyme GPX and an enhanced oxidative burden promotes endothelial dysfunction leading to DM‐related microvascular and macrovascular complications. As such, targeted antioxidant replenishment therapy using GPX‐mimetics (i.e. ebselen) may be effective in reducing the cardiovascular manifestations in disease states, such as DM.

In the present study, we showed that ebselen completely prevented endothelial dysfunction induced by CS‐exposure. We found that ebselen significantly reduced CS‐induced endothelial 3‐NT expression and that ebselen was able to prevent the loss of aortic eNOS by CS‐exposure. It is well established that under normal physiological conditions, stimulation of the vascular endothelium drives the production of NO, diffusing to the surrounding cells, in particular the underlying vascular smooth muscle cells inducing vasodilation, as well as preventing the adhesion and migration of leukocytes and platelets into/onto the arterial wall, thereby maintaining normal vascular function (Versari et al., 2009). However, vascular oxidative stress may evoke endothelial damage and disrupt vascular homeostasis (de Haan & Cooper, 2011; Versari et al., 2009). This would post significant risk for the development of CVDs and mortality. These data suggest that modulation of oxidative stress may be beneficial in the clinical treatment of CVD in the context of COPD.

Consistent with our previous studies, ebselen significantly reduced CS‐induced BALF inflammation which was largely attributed to a reduction in neutrophilic infiltration (Duong et al., 2010; Oostwoud et al., 2016). Excess neutrophils play a detrimental role in COPD particularly during periods of acute exacerbation, as they can directly induce protease‐mediated tissue damage, that has been directly correlated to worsening of emphysema in these patients (Oostwoud et al., 2016; Pesci et al., 1998). MMP activation drives a loss of lung integrity and an increase in permeability which may facilitate the spill over of proinflammatory mediators into the systemic circulation.

It was interesting to note that the whole lung gene expression of the pro‐inflammatory mediator TNFα and the oxidative stress enzyme NOX‐2, induced by CS exposure, were not reduced by ebselen pretreatment. Although not investigated in the present study, it would be worth exploring whether TNF‐α protein expression is altered following ebselen administration. Similarly, it would be worth investigating whether ebselen can directly impede the activity of the regulatory p47*phox* and other subunits of the NOX‐2 enzyme, ultimately reducing superoxide production, as this has been previously shown (Smith et al., 2012). The ROS scavenging properties of ebselen within the lung have also been established in the context of asthma, with Zhang et al. showing that following ovalbumin challenge, guinea pigs showed significantly enhanced pulmonary superoxide and hydrogen peroxide concentration, which was decreased in ebselen treated animals (Zhang et al., 2002), reinforcing the powerful antioxidant properties of ebselen in CS‐induced lung inflammation and oxidative stress.

Expression of the Gpx‐1 gene is up‐regulated in the lungs of smokers (Barnes & Celli, 2009), which may be a compensatory antioxidant mechanism in response to noxious effects of CS. Conversely, smokers and patients with established COPD have reduced Gpx activity (James & Wenzel, 2007; Versari et al., 2009; Vlahos et al., 2006), contributing to an over‐exuberant oxidative burden in the lungs of these patients (Geraghty et al., 2013). In the present study, we found that whole lung Gpx‐1 mRNA expression was significantly down‐regulated in mice exposed to CS, irrespective of ebselen treatment, further reinforcing the suggestion that loss of GPX protein would increase lung oxidative stress and inflammation. Blunted Gpx expression has also been implicated as a contributing factor in driving endothelial dysfunction, inducing apoptosis and promoting atherosclerosis systemically (Geraghty et al., 2013).

Ebselen treatment has shown promising effects on the vasculature in this study by completely preventing endothelial dysfunction in CS‐exposed mice as well as reducing BALF cellularity attributed to neutrophilic infiltration. It has been established that eNOS can undergo oxidative modification as a direct result of the heightened oxidative burden in smokers (Arunachalam et al., 2010; Edirisinghe & Rahman, 2010; Li & Forstermann, 2014; Zhang et al., 2006). Findings from this study showed that eNOS expression as quantified through immunofluorescent staining was significantly down‐regulated as a result of CS exposure. However, pretreatment with ebselen prevented CS‐induced down‐regulation of eNOS and was the most likely mechanism by which ebselen restored vascular function in CS‐exposed mice. It was also interesting to note that ebselen significantly reduced CS‐induced 3‐NT staining in the thoracic aorta indicating that ebselen completely prevented enhanced oxidative stress within the vascular endothelium, leading to sustained eNOS levels and normal vascular function in CS‐exposed and ebselen treated mice.

While it is clear from this study that ebselen can prevent CS‐induced lung inflammation and vascular dysfunction, it would be worth investigating whether ebselen can stop the progression of disease or could reverse established vascular dysfunction when administered therapeutically, that is, during established disease. We would also like to investigate the oxidative mechanisms that deplete vascular eNOS expression, as studies have shown that eNOS can become uncoupled under highly oxidative conditions (Heiss & Dirsch, 2014; Karbach et al., 2014). Direct measurement of markers of systemic inflammation including CRP or IL‐6, as well as blood leukocyte numbers would also be beneficial in the understanding of the pathology observed within the vasculature as a study by Zeng et al. has shown that serum levels of IL‐6 are significantly enhanced in patients with COPD, when compared to control subjects, although levels of CRP and TNFα remained unchanged (Zeng et al., 2019). This study has implications for people with COPD as it sheds light on the mechanisms that could explain why co‐morbid CVD is the largest killer of COPD patients and may lead to the development of novel, life‐saving therapeutics.

In conclusion, we found that chronic CS exposure in mice caused endothelial dysfunction, as a direct result of enhanced vascular oxidative stress leading to a down‐regulation of eNOS. In addition, ebselen administration significantly reduced CS‐induced lung inflammation and vascular oxidative stress leading to restored vascular endothelial function in CS‐exposed mice. Collectively, the data from the present study suggest that ebselen may be a novel therapeutic approach to the treatment of both the pulmonary manifestations and cardiovascular co‐morbidities associated with CS‐induced COPD.

## AUTHOR CONTRIBUTIONS

R.V., K.B., and S.M.H.C. did the concept and design; K.B., S.M.H.C., and H.J.S. carried out the acquisition of data. R.V., K.B., S.M.H.C., H.J.S., A.D., S.B., and S.S. did the data analysis and interpretation. All authors did the drafting, editing, and/or critical revision of the manuscript for intellectual content. R.V. also provided the resources for the work to be carried out and is the senior investigator ensuring accuracy and integrity of the work.

## CONFLICT OF INTEREST

The authors declare that they have no conflicts of interest.

## DECLARATION OF TRANSPARENCY AND SCIENTIFIC RIGOUR

This Declaration acknowledges that this paper adheres to the principles for transparent reporting and scientific rigour of preclinical research as stated in the *BJP* guidelines for Design and Analysis, Immunoblotting and Immunochemistry, and Animal Experimentation, and as recommended by funding agencies, publishers and other organisations engaged with supporting research.

## Data Availability

The data that support the findings of this study are available from the corresponding author upon reasonable request. Some data may not be made available because of privacy or ethical restrictions.
